# The Visualization of Biofilms in Chronic Diabetic Foot Wounds Using Routine Diagnostic Microscopy Methods

**DOI:** 10.1155/2014/153586

**Published:** 2014-04-15

**Authors:** Angela Oates, Frank L. Bowling, Andrew J. M. Boulton, Philip G. Bowler, Daniel G. Metcalf, Andrew J. McBain

**Affiliations:** ^1^Manchester Pharmacy School, The University of Manchester, Oxford Road, Manchester M13 9PT, UK; ^2^Department of Medicine Manchester Royal Infirmary, The University of Manchester, Oxford Road, Manchester M13 9PT, UK; ^3^ConvaTec Ltd., Deeside CH5 2NU, UK

## Abstract

Diabetic foot wounds are commonly colonised by taxonomically diverse microbial communities and may additionally be infected with specific pathogens. Since biofilms are demonstrably less susceptible to antimicrobial agents than are planktonic bacteria, and may be present in chronic wounds, there is increasing interest in their aetiological role. In the current investigation, the presence of structured microbial assemblages in chronic diabetic foot wounds is demonstrated using several visualization methods. Debridement samples, collected from the foot wounds of diabetic patients, were histologically sectioned and examined using bright-field, fluorescence, and environmental scanning electron microscopy and assessed by quantitative differential viable counting. All samples (*n* = 26) harboured bioburdens in excess of 5 log_10_ CFU/g. Microcolonies were identified in 4/4 samples by all three microscopy methods, although bright-field and fluorescence microscopy were more effective at highlighting putative biofilm morphology than ESEM. Results in this pilot study indicate that bacterial microcolonies and putative biofilm matrix can be visualized in chronic wounds using florescence microscopy and ESEM, but also using the simple Gram stain.

## 1. Introduction

The aetiological role of biofilms in diabetic wounds remains poorly understood but their formation is increasingly recognised as a potential barrier to healing [1, 2]. A limited number of studies have provided evidence for the involvement of biofilms in chronic wounds using several visualization techniques including scanning electron microscopy, epifluorescence microscopy [3–5], and confocal laser scanning microscopy (CLSM) [6–9]. No method for the identification of wound-associated biofilms has been universally recognized, partly due to the lack of unifying criteria for their identification that are applicable to a range of visualization methods.

Whilst various definitions for biofilms have been adopted, it is generally accepted that they are structured bacterial communities that are often but not always attached to surfaces and which are encased in a self-produced exopolymer matrix [10–12]. Generally, the identification of biofilms has relied on the visualization of at least two of the following three criteria: (i) microbial surface attachment [13–16], (ii) structured assemblages of microbial cells [15–17], and (iii) the presence of exopolymer matrix [14, 16]. Scanning electron microscopy and CLSM, commonly used techniques to visualize biofilms, require specialised apparatus and training and are typically found in research facilities, whereas bright-field and epifluorescence microscopy are more widely available in clinical laboratories.

The current pilot study was conducted as part of a larger study into the presence of unculturable bacteria in diabetic foot wounds [18] with the intention of addressing the commonly stated clinical requirement for a simple means of identifying biofilms in wound samples. Debridement samples (*n* = 26) were bacteriologically characterised by differential viable counting and, where sufficient sample material was available, were subjected to biofilm visualisation techniques.

## 2. Methods

### 2.1. Chemicals and Growth Media

Unless otherwise stated chemicals used were supplied by Sigma (Poole, Dorset, UK). Dehydrated bacteriological media were obtained from Oxoid (Basingstoke, Hampshire, UK) and prepared according to instructions supplied by the manufacturer.

### 2.2. Collection of Chronic Wound Tissue

This study was reviewed by the North Manchester Research Ethics Committee and the Central Manchester University Hospital Research and Development Department. Reference number: 09/H1006/41, protocol number 1.0. Twenty-six wound tissue debridement samples were collected from patients with chronic diabetic foot wounds (defined as being distal to the medial and lateral malleoli, with a known duration greater than four weeks), attending a specialist foot clinic. Wound tissue samples were taken from the wound bed and surrounding tissue using a sterile scalpel by the attending clinician and placed sterile 0.85% (w/v) saline for transportation. All samples were transported to the laboratory at 2°C and processed within 3 h of collection.

### 2.3. Differential Bacteriological Enumeration and Identification

Twenty-six tissue samples were processed as previously described [18]. Bacterial identification was based upon colony morphology, Gram staining, catalase reaction, latex coagulase reaction tests, and Lancefield group reaction to identify beta-haemolytic streptococci (Prolex Streptococcal grouping latex kits, Pro-Lab Diagnostic, Cheshire, UK) and growth on Brilliant UTI media.

### 2.4. Tissue Sectioning

Residual chronic wound tissue from four samples (which were of sufficient quantity for multiple microscopic analyses) were divided transversely (50 : 50) with a sterile scalpel and one section was embedded in optimal cutting temperature (OCT) embedding matrix and frozen at –80°C for ≥24 h. The remaining tissue sections were placed in a sterile Bijou bottle and transported immediately for ESEM imaging. To produce slide-mounted tissue sections to visualise microcolonies and biofilm architecture, OCT-embedded whole tissue samples were sectioned to a thickness of 5 *μ*m and mounted on Superfrost Plus microscope slides (Fisher Scientific, Leicestershire, UK) using a Shandon AS260 manual cryostat. Tissue sections were subjected to Gram staining and fluorescence* in situ* hybridisation (FISH).

### 2.5. Fluorescence* In Situ* Hybridisation, Fluorescent Probes, and Staining to Differentiate Bacteria, Biofilms, and Tissue

Slide-mounted tissue sections were fixed in 4% paraformaldehyde for 3 h and then subjected to a prepermeabilization step, consisting of lysozyme enzymatic buffer (100 mM Tris HCl [pH8], 50 mM EDTA, and lysozyme [5 mg/mL]), for 4 h at 45°C. Slides were then washed in wash buffer consisting of 0.9 M NaCl and 20 mM Tris and air-dried. Slides were then incubated in FISH buffer containing 50% formamide, 0.9 M NaCl, 20 mM Tris, 0.01% SDS (w/v), and 50 ng of the general eubacterial probe (EUB 338)-cy3 probe-GCT GCC TCC CGT AGG AGT [19] (Ex. 550 nm, Em. 570 nm), incubated in a humidity chamber at 55°C for 4 h and then washed with wash buffer. Once dried, slides were exposed to 10 *μ*L/mL working concentration of the carbohydrate-binding lectin, Concanavalin A conjugated Alexa Fluor 488 (Invitrogen, Paisley, UK) (Ex. 495 nm, Em. 643 nm) for 1 h at room temperature to aid in the visualization of putative biofilms. Concanavalin A binds to internal and nonreducing terminal alpha mannosyl groups, a common component of oligosaccharide of glycoproteins found in biofilms [20, 21]. Finally, to differentiate mammalian cells, the nucleic acid stain Hoechst 33252 (2 *μ*g/mL) (Ex. 350 nm, Em. 460 nm) (Sigma, Poole, Dorset, UK) was added for 1 h at room temperature [7]. All staining procedures were completed in the dark. Tissue sections were also Gram-stained as per standard protocols.

FISH images were captured using an Olympus BX51 upright microscope using a 60x and 100x objective and captured using a Coolsnap ES camera (Photometrics, AZ, USA) through MetaVue Software (Molecular Devices, CA, USA). Specific band pass filter sets for DAPI (Ex. BP365/12 nm, Em. LP397 nm), FITC (Ex. BP450–490 nm, Em. BP515–565), and Texas red (Ex. BP546/12 nm Em. LP615 nm) were used. Gram-stained images were visualized using a Zeiss Axioscop 2 microscope, Axiocam, and Axiovision Version 4.8 (Carl Zeiss Ltd., Herefordshire, UK). All images were then processed using ImageJ (http://rsb.info.nih.gov/ij).

### 2.6. ESEM of Chronic Wound Tissue

Chronic wound tissue was placed in a sterile Bijou and transported immediately for ESEM imaging. ESEM of chronic wound tissue samples was performed using a FEI Quanta 200 environmental scanning electron microscope under a low vacuum (<0.75 Torr) permitting inspection of putative biofilm structures and microcolonies whilst conserving the hydrated state of the sample.

## 3. Results

### 3.1. Viable Bacterial Counts from Wound Samples

**Figure 1:**
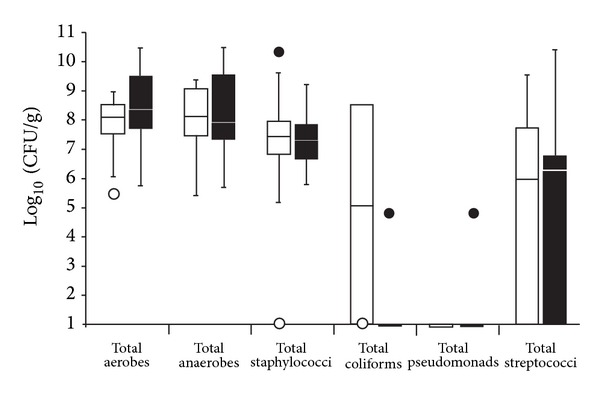
Differential viable counts of selected bacterial groups from 26 chronic wound samples. The lower and upper boundaries of the boxes represent quartiles 1 and 3, respectively, and horizontal bars within the boxes represent median values. ○ represents minimum outliers and ● the maximum outliers. White bars represent samples from which pathogens [18] were not isolated and black bars samples from which pathogens were isolated.

All 26 tissues samples harboured aerobic and facultative anaerobic species at cell densities equal to or greater than 5 log_10_ CFU/g of tissue, as shown in Figure 1 and Table 1. Of the four samples which were subjected to visualisation techniques, the total anaerobic, aerobic and viable counts of staphylococci were within two standard deviations of the mean of the total populations sampled. Samples 1 and 2 harboured streptococci and Sample 4 was the only tissue sample from which coliforms were isolated, (Table 1).

### 3.2. Biofilm Visualization

**Figure 2:**
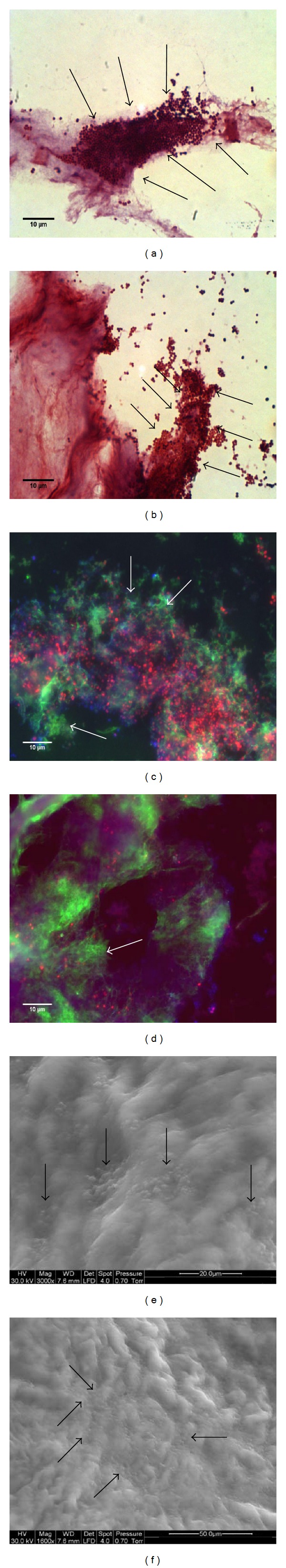
Images acquired from Sample 1. (a) and (b) are replicate images from Gram-stained sections; (c) and (d) (replicates) have been visualized using a combination of FISH (red), to indicate eubacteria, ConA (green) to indicate biofilm matrix, and other ConA-reactive material, and with Hoechst 33252 (blue) for the detection of nucleic acids. (e) and (f) show replicate ESEM images. Presumptive bacterial microcolonies and biofilm matrix have been indicated by arrows.

**Figure 3:**
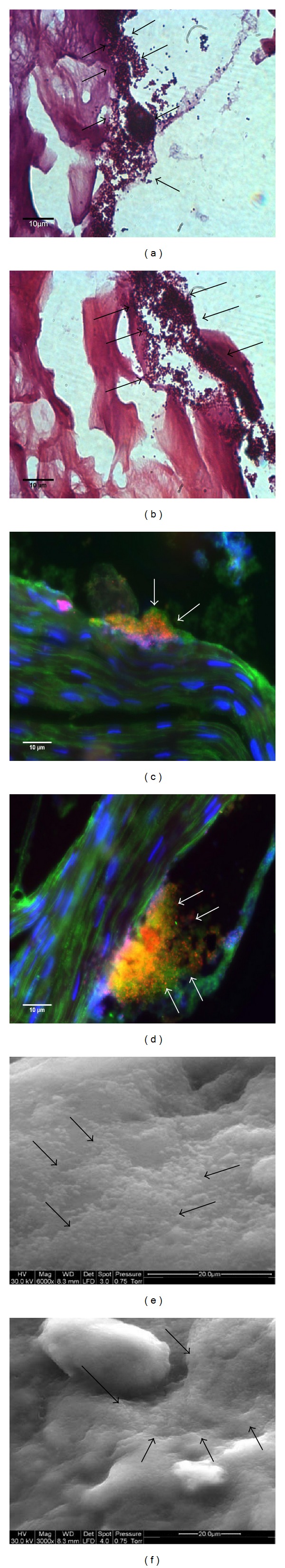
Images acquired from Sample 2. See legend to Figure 1.

**Figure 4:**
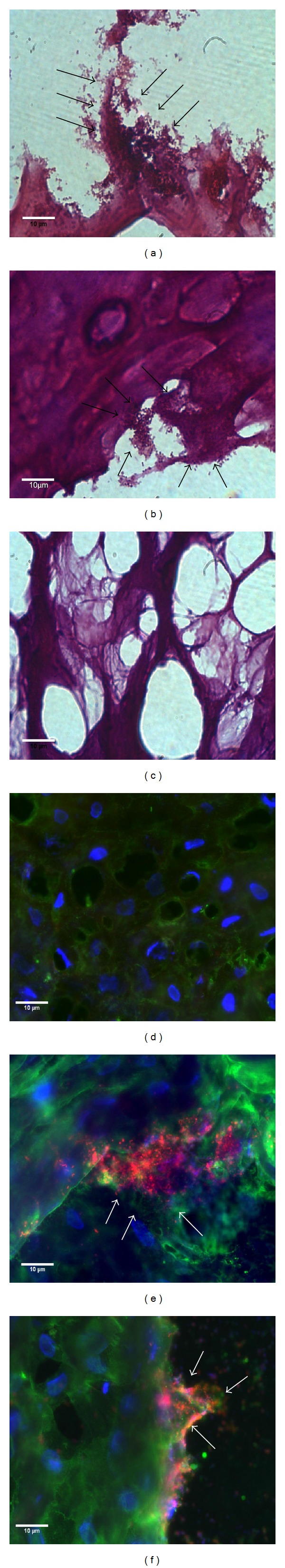
Images acquired from Sample 3. See legend to Figure 1.

**Figure 5:**
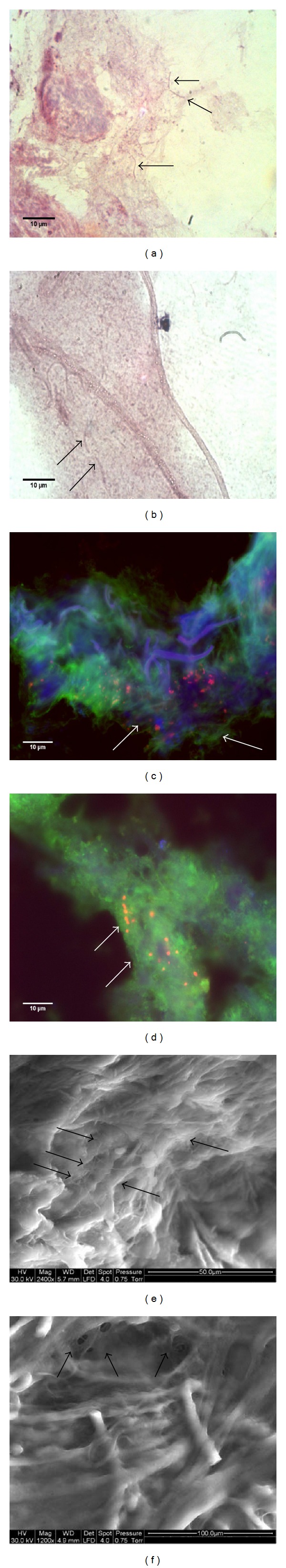
Images acquired from Sample 4. Putative biofilm matrix is indicated by arrows. See legend to Figure 1.

Figure 2 shows the stained and imaged chronic wound sections from Sample 1. Gram staining, the use of a eubacterium specific FISH probe Figures 2(c)-2(d), and ESEM Figures 2(e)-2(f) analysis indicate the presence of bacterial microcolonies embedded within and/or upon surfaces of wound tissue.  Figure 3 similarly shows imaged chronic wound sections from Sample 2, as does Figure 4 for Sample 3. Bacterial infiltration into internal portions of the tissues tissue sections is not apparent Figures 4(c)-4(d). Images derived from Sample 4 are shown in Figure 5. Whilst bacteria have been indicated in this sample by FISH, Gram staining showed only putative bacterial cells and, furthermore, discrete bacterial cells were not revealed by in ESEM. It is possible, however, that, in the latter case, cells may have been obscured by biofilm matrix material.

Based on localization of reactive material, the utility of ConA and Hoechst 33252 as specific biofilm indicators is limited by the reactivity of structures associated with host cells. However, it is likely that Con-A-reactive material adjacent to bacterial microcolonies (as indicted by the FISH probe) is biofilm matrix. This is particularly evident in Figures 2 and 3.

## 4. Discussion

The taxonomically diverse microbial communities which occur in diabetic foot wounds may include both aerobic and anaerobic organisms many of which are potentially pathogenic [22, 23]. The role that such organisms play in impeding healing has been previously documented [22–26]. However, the aetiological role of bacterial biofilms in diabetic foot wounds remains poorly understood, although they are becoming recognised as a potential impediment to healing [1, 2]. As such, there is an increasing clinical need to identify biofilms in these wounds. In the current study, quantitative and diagnostic culture techniques were used to measure wound bioburdens, whilst tissue samples were also subjected to bright-field, epifluorescence, and ESEM to identify structures associated with the biofilm phenotype. Each method was selected to represent biofilm visualization methods commonly reported in the literature and their presumed ability to identify at least two of the following three criteria: (i) microbial surface attachment, (ii) structured assemblages of microbial cells, and (iii) the presence of exopolymer matrix. According to previous reports, images of sections stained using hematoxylin and eosin, and Gram-stained biofilms, for example, readily may reveal microcolonies attached to tissues but reportedly fail to recognize the exopolymer matrix [5, 27]. The application of epifluorescence microscopy and CLSM however allows for the specific staining of the exopolymer matrix [21, 28], but not all studies using fluorescence microscopy have adopted this approach [7, 25].

A limitation of biofilm matrix staining using a carbohydrate marker such as the (fluorescently labeled) carbohydrate-binding lectin, Concanavalin-A, is the fact that reactive materials are also commonly associated with mammalian cells. It is, therefore, important to consider the location of reactive material. The feasibility of this approach may be enhanced by using FISH-probes for bacteria and a nucleic acid stain such as Hoechst 33252.

When exploring biofilms using scanning electron microscopy, a high level of resolution and detail can be obtained, potentially revealing biofilm-specific morphology, but also individual cells and their spatial location. Exopolymer matrix is amorphous material which may appear as a layer covering the biofilm, or as a fibrous material. Preparation of the sample for SEM involves dehydrating the sample which can affect the overall morphology of the biofilms and the appearance of the biofilm matrix. These considerations can partially be overcome with the application of ESEM or cryo-SEM which preserve the hydrated state of the biofilm.

In the present study, examination of slide-mounted, Gram-stained tissue sections revealed microcolonies attached to tissues which are indicative of the biofilm phenotype. These microcolonies comprised Gram-positive cocci (Samples 1–3), which corresponds to the organisms isolated by culture, coagulase negative staphylococci (Sample 1), and* Staphylococcus aureus* from Samples 2 and 3. Fluorescence microscopy of slide mounted tissue sections using FISH produced images which generally agreed with those obtained using Gram staining, such as microcolonies in Samples 1–3 and the low numbers of bacilli in Sample 4. An additional benefit of fluorescence microscopy is the option to detect biofilm matrix using a fluorescent probe. All four samples showed evidence of exopolymer matrix using the Concanavalin A conjugated Alexa Fluor 488 probe. Biofilm matrix was identified as distinct material encasing stained bacterial cells. Interestingly, combining tissue sectioning and simple staining techniques, microcolonies and bacterial exopolymer could be readily identified, with the extent of bacterial infiltration discernible from the depth of each slide section. Whilst evidence of biofilm involvement could be found in all tissues examined, sectional analysis of tissues suggested that biofilms were limited to surface tissue sections with little or no evidence of bacteria or matrix found at depths greater than 20 *μ*m.

The tissue samples were imaged further using ESEM. To conserve their hydrated state inspection of tissue surfaces was performed using an ESEM under a low vacuum (<0.75 Torr). Whilst ESEM is a method which requires access to the specialised equipment and training to ensure conservation of biofilm architecture and tissues, the images generated generally agreed with those gathered using the less complex methods using Gram and fluorescent staining, with microcolonies and/or amorphous substances (indicative of biofilms) identified in all samples.

The three visualisation techniques involved staining, fluorescence, and high-resolution microscopy to identify structures typical of the biofilm phonotype. Whilst the data presented represent a relatively small sample size, the outcomes of each method were broadly congruent. Since each method detects at least two of the three criteria (i) microbial surface attachment [13–16], (ii) structured assemblages of microbial cells [15–17], and (iii) the presence of exopolymer matrix [14, 16], biofilms can be detected and, perhaps more significantly, this can be achieved using techniques such as Gram staining and fluorescent microscopy which are comparatively cost effective and simple to conduct, requiring equipment that can be found in many diagnostic laboratories.

The growing interest in the role biofilms play in chronicity and impaired healing of diabetic wounds has led to an increased clinical requirement for a simple means of identifying biofilms in wound samples. More readily available methods such as Gram staining and bright-field microscopy can efficiently detect microcolonies associated with the biofilm phenotype and may therefore be of use for the identification of biofilms where expediency and cost-effectiveness are required.

## Figures and Tables

**Table 1:** Differential viable counts of selected bacterial groups from the four imaged samples.

Sample	Total:				
	Aerobic count	Anaerobic count	Staphylococci	Coliforms	Streptococci
1	10.31	9.07	8.93	ND	7.73
2	10.46	10.49	5.93	ND	10.40
3	7.28	7.33	7.24	ND	ND
4	8.39	8.36	ND	8.49	ND
Mean*(*n* = 26)	8.15 (1.30)	7.87 (1.72)	7.09 (1.77)	3.20 (4.27)	3.37 (3.90)

Values are log_10_⁡CFU/g. Samples 1–4 correspond to the numbered imaged samples in Figures 2–5. *refers to mean values from the 26 samples for which full data is presented in Figure 1 (standard deviations are given in parentheses). *Pseudomonas  *was not detected in samples 1–4 and was only detected in one of the 26 samples (at 4.7 log_10_, ⁡CFU/g).
